# Capture and Prediction of Rainfall-Induced Landslide Warning Signals Using an Attention-Based Temporal Convolutional Neural Network and Entropy Weight Methods

**DOI:** 10.3390/s22166240

**Published:** 2022-08-19

**Authors:** Di Zhang, Kai Wei, Yi Yao, Jiacheng Yang, Guolong Zheng, Qing Li

**Affiliations:** National and Local Joint Engineering Laboratories for Disaster Monitoring Technologies and Instruments, China Jiliang University, Hangzhou 310018, China

**Keywords:** rainfall-induced landslide, attention mechanism, entropy weight methods, an attention-based temporal convolutional neural network, landslide hazard degree

## Abstract

The capture and prediction of rainfall-induced landslide warning signals is the premise for the implementation of landslide warning measures. An attention-fusion entropy weight method (En-Attn) for capturing warning features is proposed. An attention-based temporal convolutional neural network (ATCN) is used to predict the warning signals. Specifically, the sensor data are analyzed using Pearson correlation analysis after obtaining data from the sensors on rainfall, moisture content, displacement, and soil stress. The comprehensive evaluation score is obtained offline using multiple entropy weight methods. Then, the attention mechanism is used to weight and sum different entropy values to obtain the final landslide hazard degree (LHD). The LHD realizes the warning signal capture of the sensor data. The prediction process adopts a model built by ATCN and uses a sliding window for online dynamic prediction. The input is the landslide sensor data at the last moment, and the output is the LHD at the future moment. The effectiveness of the method is verified by two datasets obtained from the rainfall-induced landslide simulation experiment.

## 1. Introduction

Rainfall-induced landslides are geological hazards triggered by prolonged rainfall or short-term heavy rainfall. Scholars have conducted in-depth research on landslide susceptibility mapping [1], data modeling [2], and mechanism analysis [3].

Machine learning (ML) and deep learning (DL) are important methods for landslide prediction because of their ability to achieve complex nonlinear modeling. Many ML and DL methods are used for landslide detection and prediction with better performance than traditional methods. Wei et al. proposed an attention-constrained neural network with overall cognition (OC-ACNN) to capture features to predict landslides [4]. Ghorbanzadeh et al. used different deep convolutional neural networks (CNNs) for landslide remote sensing images and achieved better results in landslide mapping [5]. An integrated framework of DL models with rule-based object-based image analysis (OBIA) to detect landslides was explored by Ghorbanzadeh et al. [6]. Wang et al. optimized the Elman neural network with the genetic algorithm and used it to implement the prediction of landslide displacement [7]. Wang et al. compared five machine learning methods for reservoir displacement prediction, and the Hodrick–Prescott filter decomposed the cumulative displacement into trend displacement and periodic displacement [8]. Wang et al. predicted the intrinsic evolution trend of landslide displacement by (double exponential smoothing, DES) DES-VMD-LSTM, based on the Gaussian process regression (GPR) model to assess the uncertainty in the first prediction [9]. Miao et al. applied the fruit fly optimization algorithm back-propagation neural network (FOA-BPNN) for the prediction of random displacements [10]. Gong et al. considered the problem of interval prediction of landslide displacements and proposed a new method of interval prediction of landslide displacements combining dual-output least squares support vector machine (DO-LSSVM) and particle swarm optimization (PSO) algorithms [11]. Time series analysis and long short-term memory neural networks are used in landslide displacement prediction [12,13]. Lin et al. analyzed the internal relationship between rainfall, reservoir water level, and periodic landslide displacement and used the double-bidirectional long short-term memory (Double-BiLSTM) model to predict landslide displacement [14]. Zhang et al. proposed a method based on Gated Recurrent Unit (GRU) and Fully Integrated Empirical Decomposition of Adaptive Noise (CEEMDAN) for the dynamic prediction of landslide displacement [15]. The application of hybrid methods based on metaheuristics (MH) in the field of geohazards is a recent research direction in disaster prediction. Ma et al. conducted a comparative study on MHs and proposed a new hybrid algorithm, namely MH-based support vector machine regression (SVR) [16]. The hybrid method has high performance in terms of accuracy and reliability for landslide displacement prediction. Meanwhile, the hybrid method combined with a multiverse optimization (MVO) for hyperparameter optimization of MHs [17] improves the reliability of disaster prediction modeling.

Rainfall is commonly used for early warning as an important trigger for landslides. Cost-sensitive rainfall thresholds were investigated by Sala et al. and sensitivity analysis was performed [18]. However, rainfall thresholds that are difficult to standardize cannot be used as early warning signals for the occurrence of landslides. Changes in soil moisture are an important factor in landslides. Domínguez-Cuesta et al. focused on the role of rainfall and soil moisture as triggering and evolutionary factors for unstable events [19]. Soil moisture saturation and sudden rainfall are more likely to lead to landslides. Chen et al. analyzed the role of soil moisture index (SWI) in landslides based on 279 mass movements that occurred in Taiwan during 2006–2017 [20].

These data-driven approaches effectively implement the displacement prediction problem for landslides; however, these models do not consider correlations among multiple sensor data and do not capture warning signals in sensor data well. Entropy value, as a physical quantity describing the degree of data chaos, has also been used to analyze landslide risk [21]. However, landslide hazard analysis using the information entropy value method does not take into account the effects of different entropy values on landslide sensor data. A single entropy value method for landslide warning feature analysis failure will result in the possibility of misclassification.

*Challenges:* First, there are many landslide monitoring sensors, but the methods of effectively capturing warning signals are less studied. Second, there are correlations among different types of landslide sensor data, which need to be analyzed. Third, the accuracy of data-driven rainfall-induced landslide hazard prediction models needs to be improved.

*Contributions*:

- We combine an attention mechanism with multiple entropy weight methods and propose an attention-fusion entropy weight method (En-Attn) to capture warning signals based on massive landslide sensor data.
- We propose an attention-based temporal convolutional neural network for landslide warning signals prediction based on massive sensor data.
- We carry out the experimental simulation of rainfall-induced landslides, collect sensor data when landslides occur, analyze the precursory warning characteristics of the data, and use a variety of entropy weight methods to analyze the characteristics of warning signals offline.
- Our model is validated on two datasets obtained from rainfall-induced simulation experiments, and our model has high accuracy compared with similar landslide warning capture and prediction methods.

## 2. Methods

### 2.1. Capture Models of Landslide Warning Signal

We obtain massive sensor data from landslide simulation experiments, including rainfall, the soil moisture content in shallow layers, the soil moisture content in deep layers, soil stress, and displacement. The evaluation of landslide warning signals is to extract the warning features from these massive sensor data to characterize the landslide warning situation. The entropy weight methods (EWM) can be used to assess the degree of landslide hazard [21].

#### 2.1.1. Entropy Weight Methods

Entropy is a measure of uncertain information. The smaller the entropy value, the greater the amount of information and the greater the weight. The entropy weight method (EWM) [22] is an objective weighting method. The canonical EWM uses information entropy (*InEn*) [23] as the basis for calculation. In fact, there are many entropy methods, namely approximate entropy [24], sample entropy [25], fuzzy entropy [26], and permutation entropy [27]. Therefore, an improved entropy method can be obtained by replacing the information entropy in the canonical entropy weight method with the following four entropy values: approximate entropy (*ApEn*), sample entropy *(SampEn*), fuzzy entropy (*FuzzyEn*), permutation entropy *(PeEn*).

The calculation process of the EWM [28] has five steps.

Step 1: Data normalization using Equation (1).

Step 2: Calculate the entropy value using Equation (2).

Step 3: Calculate the coefficient of variation using Equation (3).

Step 4: Calculate weights using Equation (4).

Step 5: Calculate the entropy weight score using Equation (5).

(1) $$x_{ij}=z_{ij}/\sum_{i=1}^{N}z_{ij}$$

(2) $$e_{j}=f_{En}(x_{ij}),i\in [1,N],e_{j}\in [0,1]$$

(3) $$d_{j}=1-e_{j}$$

(4) $$\omega_{j}=d_{j}/\sum_{j=1}^{N}d_{j}$$

(5) $$s_{i}=\sum_{j=1}^{M}\omega_{j}x_{ij},i=1,2,\cdots ,N$$

where 

$z_{ij}$ is the raw data at row *i* and column *j* in the sensor dataset.

$x_{ij}$ is the data normalized by $z_{ij}$.

$e_{j}$ is the entropy value of $x_{ij}$.

$f_{En}$ is the method for calculating the entropy values using Equations (6)–(26) for the specific formula.

N is the number of rows in the sensor dataset.

$d_{j}$ is the coefficient of variation of $x_{ij}$.

$\omega_{j}$ is the corresponding weight of each column of data obtained by the EWM.

$s_{i}$ is the weight entropy score.

M is the number of columns in the sensor dataset.

Information entropy (*InEn*) [23] can be calculated by Equation (6).

(6) $$f_{InEn_{j}}=-\frac{1}{\ln N}\sum_{i=1}^{N}x_{ij}\ln x_{ij},e_{j}\in [0,1]$$

where

ln denotes the natural logarithm.

$f_{InEn_{j}}$ denotes the information entropy value.

The calculation of *ApEn* can also be understood as the degree of self-similarity of a sequence in the pattern. For the change of a signal sequence, the change of the approximate entropy value can be used to achieve the purpose of effective identification. The biggest advantage of the approximate entropy calculation is that it does not require a large amount of data, most of the measured time series can meet the requirements, and the obtained results are robust and reliable [29].

The calculation of approximate entropy (*ApEn*) is as follows:

(7) $$\begin{array}{l} X_{i}=[x(i),x(i+1),\cdots ,x(i+m-1)] \end{array}$$

(8) $$d[X_{i},X_{j}]=\max|x(i+k)-x(j+k)|,k\in (0,m-1)$$

(9) $$B_{i}(r)=num\{d[X_{i},X_{j}]<r\}$$

(10) $$\Phi_{i}^{m}(r)=\frac{B_{i}}{N-m+1}$$

(11) $$f_{ApEn}=\Phi^{m}(r)-\Phi^{m+1}(r)$$

where

$d[X_{i},X_{j}]$ denotes the distance between the vector *X_i_* and *X_j_*.

$B_{i}$ is the number of items that satisfy the condition $d[X_{i},X_{j}]<r$.

r denotes the similarity tolerance threshold.

$\Phi_{i}^{m}$ denotes the ratio of the approximate quantity to the total quantity, namely the approximate ratio.

$f_{ApEn}$ denotes the approximate entropy value of sequence *X_i_*.

m is the dimension of *X_i_*, which is an artificially set parameter value.

*ApEn* characterizes the complexity of a sequence. The value of *ApEn* is less affected by the amount of data and is suitable for non-stationary and nonlinear sequences. *ApEn* preserves the time series information in the original signal sequence and reflects the characteristics of the signal sequence on the structural distribution. The entropy value of the fault signal will be greater for fault data present in a set of continuous data, so *ApEn* is often used to detect the fault signal. The fault signal here refers to the presence of multiple abnormal signals in a set of sequential signals.

*SampEn* is an improved method based on *ApEn* [29]. The *SampEn* has better consistency. If one time series has a higher *SampEn* value than another time series, then the other *r* and *m* values also have higher *SampEn* values. Meanwhile, *SampEn* is not sensitive to missing data [29].

The calculation of sample entropy (*SampEn*) is as follows:

(12) $$B_{i}^{m}(r)=\frac{1}{N-m}num\{d[X_{i},X_{j}]<r\}$$

(13) $$B^{m}(r)=\frac{1}{N-m+1}\sum_{i=1}^{N-m+1}B_{i}^{m}(r)$$

(14) $$f_{SampEn}=-\ln(B^{m+1}(r)/B^{m}(r))$$

where

$B_{i}^{m}$ denotes the ratio of the number of $d[X_{i},X_{j}]<r$ to the total number of vectors *N*-*m*, for a given threshold *r* (*r* > 0).

$f_{SampEn}$ denotes the sample entropy value of the sequence *X_i_*.

In the definitions of *ApEn* and *SampEn*, the similarity of vectors is determined by the difference in absolute values of the data. Correct analysis results cannot be obtained when there are slight fluctuations in the data used or baseline drift. *FuzzyEn* removes the influence of baseline drift through mean operation, and the similarity of vectors is no longer determined by the absolute amplitude difference, but determined by the shape of the fuzzy function determined by the exponential function, thereby fuzzifying the similarity measure [26]. The *FuzzyEn* uses an exponential function to fuzzify the similarity measurement formula. The continuity of the exponential function makes the fuzzy entropy change continuously and smoothly with the parameter change.

The calculation of fuzzy entropy (*FuzzyEn*) is as follows:

(15) $$Y_{i}=[x(i),x(i+1),\cdots ,x(i+m-1)]-x_{0}(i),i=1,2,\cdots ,N-m+1$$

(16) $$x_{0}(i)=\frac{1}{m}\sum_{j=0}^{m-1}x(i+j)$$

(17) $$d_{i,j}^{m}=d[Y_{i},Y_{j}]=\underset{k\in (0,m-1)}{\max}|x(i+k)-x_{0}(i)-x(j+k)-x_{0}(j)|$$

(18) $$D_{i,j}^{m}=\exp\left[-\frac{{\left(d_{i,j}^{m}\right)}^{n}}{r}\right]$$

(19) $$\psi^{m+1}(r)=\frac{1}{N-m+1}\sum_{i=1}^{N-m+1}\left(\frac{1}{N-m}\sum_{j=1,j\neq i}^{N-m+1}D_{i,j}^{m}\right)$$

(20) $$f_{FuzzyEn}=-\ln(\psi^{m+1}(r)/\psi^{m}(r))$$

where

*m* denotes the embedding dimension.

Y denotes the sequence after the phase space reconstruction of *X*.

$x_{0}$ is the mean of m consecutive x(i+j).

$d_{i,j}^{m}$ denotes the maximum value of the difference between the corresponding endpoints of *Y_i_* and *Y_j_*.

$D_{i,j}^{m}$ is the similarity between *Y_i_* and *Y_j_* after using the fuzzy membership function.

$\psi^{m}$ is a function defined like $\Phi_{i}^{m}$ and $B_{i}^{m}$.

$f_{FuzzyEn}$ denotes the fuzzy entropy value of sequence *X_i_*.

Permutation entropy (*PeEn*) is a method to detect the randomness and dynamic mutation behavior of time series. The *PeEn* has the characteristics of simple and fast calculation, strong anti-noise ability, and can realize the characteristics of online monitoring of mutation signals. *PeEn* introduces the idea of permutation when calculating the complexity between reconstructed subsequences.

The calculation of permutation entropy (*PeEn*) is as follows:

(21) $$Y_{i}=[x(i),x(i+\tau ),\cdots ,x(i+(m-1)\tau )],i=1,2,\cdots ,N-m+1$$

(22) $$x(i+(j_{1}-1)\tau )\leq x(i+(j_{2}-1)\tau )\leq \cdots \leq x(i+(j_{m}-1)\tau )$$

(23) $$S(l)=(j_{1},j_{2},\cdots ,j_{m}),l=1,2,\cdots ,k,and\;k\leq m!$$

(24) $$P_{i}=\frac{Number(Y_{i})}{N-(m-1)\tau }$$

(25) $$PE(m)=-\sum_{i=1}^{k}\left(P_{i}\ln P_{i}\right)$$

(26) $$0\leq f_{PeEn}=PE/\ln(m!)\leq 1$$

where

*m* denotes the embedding dimension.

*τ* denotes the time delay factor.

k=N−(m−1)τ,j=1,2,⋯,k

S is a set of symbol sequences consisting of the index of each element position column after each reconstructed component is rearranged in ascending order.

$j_{m}$ is the column index of the position of the *m*th element in the vector.

$P_{i}$ is the probability of occurrence of each sort.

PE denotes the permutation entropy value of the sequence.

$f_{PeEn}$ denotes the normalized value of the permutation entropy.

The matrix has *k* reconstruction components in total, and each reconstruction component has *m*-dimensional embedded elements. Arrange the *j*th category in the matrix in ascending order according to the size of the array using Equation (22). 

$j_{1},j_{2},\cdots ,j_{m}$ represents the subscript index value of each element in the reconstructed component. Note that the above sequence has a parameter *τ*, namely the time delay factor, which must be a positive integer. In fact, this parameter can be understood as the downsampling of the sequence. For example, when *τ* = 3, it is sampling every three data points. When *τ* = 1, the sequence is the same as the sequence definition of the *ApEn* and *SampEn*.

#### 2.1.2. Attention-Fusion Entropy Method

**Figure 1:**
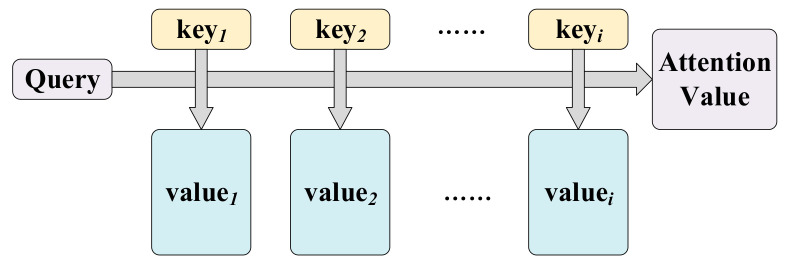
Overview of attention mechanism.

The attention mechanism can pay attention to important parts of the sequence data [2,30]. Queries and key-value pairs are mapped to outputs. The calculation process of the attention mechanism is shown in Figure 1.

Equation (27) shows the score function, and Equation (28) shows the attention calculation process. The score function is essentially seeking a degree of similarity, and the *Softmax* function is to normalize the weights at all positions so that the sum is equal to one [31].

(27) $$f(Q,K)=\frac{Q^{T}K}{\sqrt{d}}$$

(28) C=Attention(Q,K,V)=Softmax(f(Q,K))V

where

*Q* denotes the queries, and $Q=W^{q_{i}}X_{t}$*,* where $W^{q_{i}}$ is the weight corresponding to *Q.*

*K* denotes the keys $K=W^{k_{i}}X_{t}$, where $W^{k_{i}}$ is the weight corresponding to *K*.

*V* denotes the values $V=W^{v_{i}}X_{t}$, where $W^{v_{i}}$ is the weight corresponding to *V*.

*C* denotes the result of the weighted summation of weights and variables.

$\frac{1}{\sqrt{d}}$ denotes the scaling factor.

The role of the scaling factor is to keep the dot product of *Q* and *K* from becoming too large [31]. Once the dot product is too large, the activation function *Softmax* enters a region with a small gradient. The attention mechanism is used for the calculation to fuse multiple EWMs, and the fused entropy method is obtained, which is named as En-Attn.

**Figure 2:**
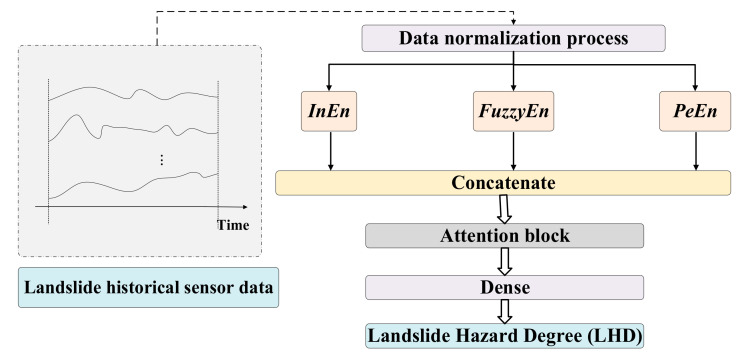
Overview of an attention-fusion entropy weight method (En-Attn).

Figure 2 shows that the input of the En-Attn model is historical sensor data, including rainfall, shallow moisture content, deep moisture content, displacement, and soil stress. The three types of data are calculated by three EWMs for comprehensive evaluation scores. The difference between these three entropy weight methods is that the entropy is different, namely *InEn*, *FuzzyEn*, and *PeEn*. The reason why *ApEn* and *SampEn* are not used in the En-Attn model is that *FuzzyEn* is an improvement on *SampEn* and *ApEn*. Meanwhile, in the actual dataset, the difference between these three methods is not obvious. For the same datasets, the result of getting almost the same output needs to be computed three times, which consumes computation time and occupies the memory of the computation space. Therefore, *FuzzyEn* is chosen instead of the three EWMs to reduce the time and space complexity of the En-Attn method. The demonstration of the details of these three EWMs for landslide sensor data processing is presented in Section 4.1.

The attention mechanism is used to fuse the outputs of the three EWMs (*InEn*, *FuzzyEn*, and *PeEn*) and finally outputs landslide hazard degree (LHD). Algorithm 1 elaborates the specific calculation steps.
**Algorithm 1:** Attention-fusion entropy weight method (En-Attn).Initialization: M, *m*, *r*, *d*, *W* Input: the raw data z **Entropy weight methods** For *j* = 1:M Data normalization using Equation (1). Calculate *InformEn* using Equation (6). Calculate *FuzzyEn* using (15)~(20). Calculate *PeEn* using (21)~(26). Calculate the coefficient of variation using Equation (3). Calculate weights using Equation (4). Obtain the entropy weight scores using Equation (5). End if Output: $S_{InEn},S_{FuzzyEn},S_{PeEn}$ **Attention calculation** $Q=K=V=W\cdot [S_{InEn},S_{FuzzyEn},S_{PeEn}]$ $S_{En-Attn}=Softmax(\frac{Q^{T}K}{\sqrt{d}})V$ $\mathrm{LHD}=normalize(S_{En-Attn})$ Output: LHD.

### 2.2. Prediction Model of Landslide Warning Signal

The prediction model of the hazard degree of rainfall-induced landslides is based on temporal convolutional neural networks (TCNs). TCNs have a good predictive effect on the processing of time series data [32,33]. We add an attention module to the data before TCN input to extract the prediction features of the input data; we also add an attention module to the output data of TCN to extract the features of the output data to improve the performance of TCN.

**Figure 3:**
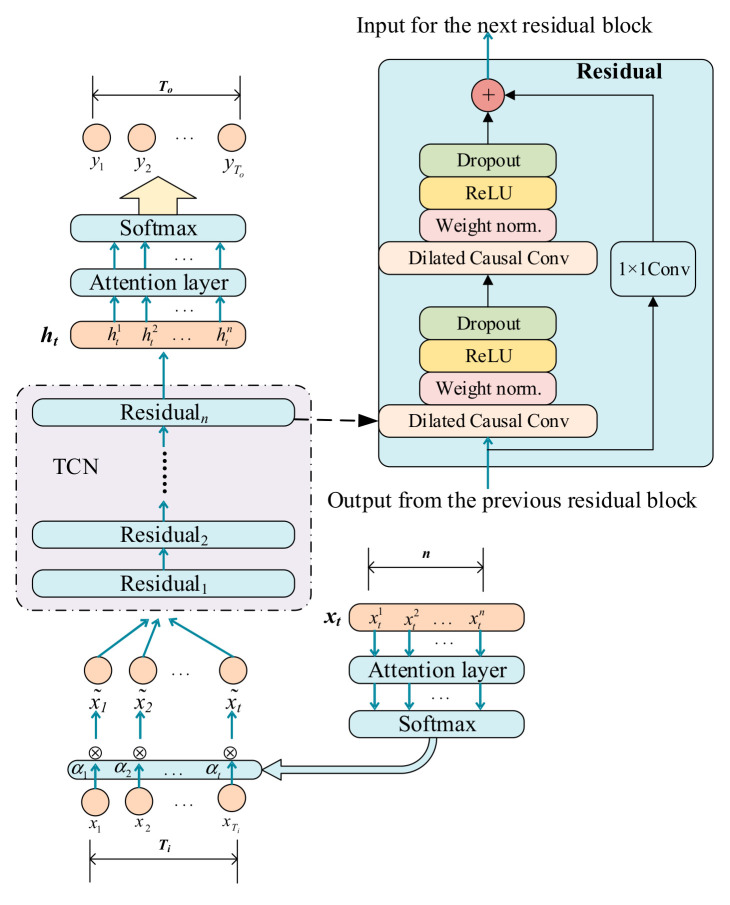
The overall framework of the attention-based temporal convolutional neural network (ATCN).

The TCN incorporating the attention mechanism is shown in Figure 3, including the attention mechanism (I-Attn) in the input stage, the attention mechanism (T-Attn) after the TCN output, and the TCN that plays the main prediction role. The input of I-Attn is sensor data at time *t* and the hidden layer at time *t* − 1, and the output is the attention weight at time *t*. The input of T-Attn is the hidden layer at time *t*, and the output is the size of the attention weight at time *t* and the weight value of the TCN’s output, which is the final predicted output value. TCN is composed of multiple residual blocks [32]. The output of the previous residual block is the input of the next residual block. The 1D convolution in TCN enables equal lengths of the input and output sequences [34]. Causal convolution ensures that the prediction process does not suffer from data leakage. TCN enlarges the convolutional field size, which can be obtained from Equation (29). The calculation of the number of residual blocks is obtained from Equation (30).

(29) $$r=1+\sum_{i=0}^{n-1}2(k-1)b^{i}=1+2(k-1)\frac{b^{n}-1}{b-1}$$

(30) $$n=\left[\log_{b}\left(\frac{\left(l-1)(b-1\right)}{2(k-1)}+1\right)\right]$$

where

*k* denotes the size of the convolutional kernel.

*B* denotes the size of the dilated base.

*N* denotes the number of residual blocks.

*L* denotes the length of the input tensor.

**Figure 4:**
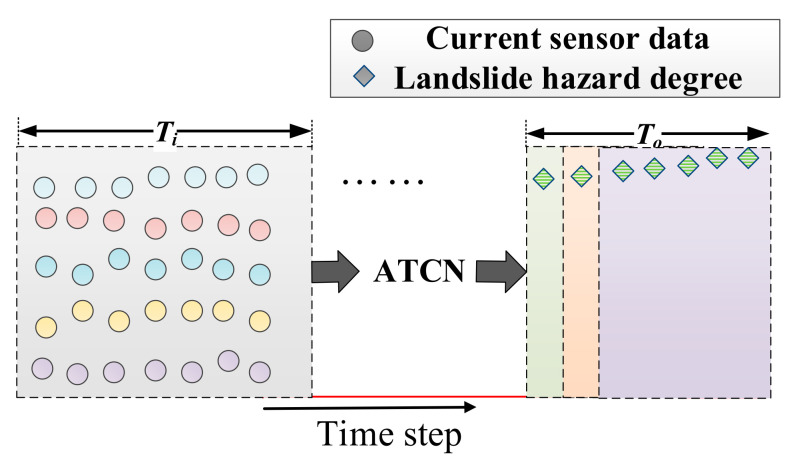
Sliding window for dynamic prediction of sensor data.

In the actual landslide experiment, the sensor data are transmitted back to the host computer as a continuous string of arrays. The dynamic sliding prediction of the ATCN model is implemented using a sliding window as a way to process the dynamic data, as shown in Figure 4. The input of the sliding window is the five-dimensional sensor data of *T_i_* length, and the output is the landslide hazard degree (LHD) of *T_o_* length. The sliding window moves forward with the time step while the predicted value is output. Algorithm 2 illustrates the specific steps of the landslide warning signals prediction model (ATCN). The performance of the ATCN is experimentally verified in Section 4.2.
**Algorithm 2:** Attention-based temporal convolutional neural network (ATCN).Input: $x_{t}=\{x_{t}^{1},x_{t}^{2},\cdots ,x_{t}^{T_{i}}\}$ Data normalization using Equation (1). **I-Attn calculation:** $Q_{i}=K_{i}=V_{i}=W_{i}\cdot x_{t}$ ${\tilde{x}}_{t}=Softmax(\frac{Q_{i}{}^{T}K_{i}}{\sqrt{d_{i}}})V_{i}$ **Predictor:** $h_{t}=f_{TCN}({\tilde{x}}_{t})$ **T-Attn calculation:** $Q_{o}=K_{o}=V_{o}=W_{o}\cdot h_{t}$ $y_{t}=Softmax(\frac{Q_{o}{}^{T}K_{o}}{\sqrt{d_{o}}})V_{o}$ Output: $y_{t}=\{y_{t}^{1},y_{t}^{2},\cdots ,y_{t}^{T_{o}}\}$ Update $x_{t}\leftarrow x_{t+1}$, and repeat the above steps.

## 3. Data Acquisition and Processing

### 3.1. Landslide Simulation Platform

**Figure 5:**
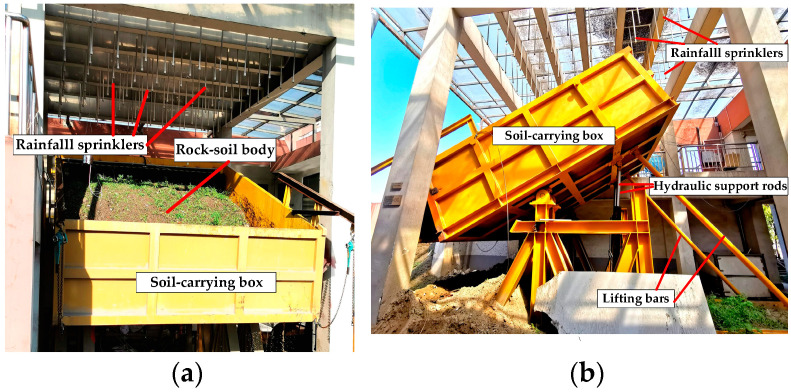
Landslide simulation platform (LSP). (**a**) Main view of the LSP; (**b**) Side view of the LSP.

The landslide simulation platform (LSP) is built to simulate the occurrence of rainfall-induced landslides. The landslide simulation platform (LSP) simulates a small monitoring area in a mountain rather than a large area such as a natural landslide itself. This is because simulating a mountain in nature is actually very challenging, and all we can do is simulate a certain monitoring area. In nature, multiple monitoring zones work together on a large mountain. The analysis of a monitoring zone is a prerequisite for data analysis and early warning of a large mountain. Figure 5 shows the physical objects of the LSP. The structure of the LSP includes the simulated rainfall system and the sensor measurement system.

The simulated rainfall system consists of the following components: rainfall sprinklers, soil-carrying box, hydraulic support rods, and lift bars. The rain sprinklers simulate the natural rainfall environment, and controlling the amount of rainfall can simulate the rainstorm. The soil-carrying box contains rock and soil mass to simulate natural slope conditions. The hydraulic support rods and the lifting bars can adjust the angle of the soil-carrying box to simulate the angle of the potential landslide body in nature. Water will seep out of the tube wall as it passes through the porous ceramic tube, simulating underground water in the rock and soil mass.

The experimental process includes five steps:

Step 1: Place the rock and soil mass inside the soil box.

Step 2: Install five types of sensors at the appropriate positions.

Step 3: Use the hydraulic support rod to adjust the soil box to a suitable angle. Here, we chose 30°.

Step 4: Turn on the rain sprinklers for rainfall simulation and use the monitoring software to monitor the sensor data and save it to the database.

Step 5: Analyze and process the sensor data after the experiment is completed.

**Figure 6:**
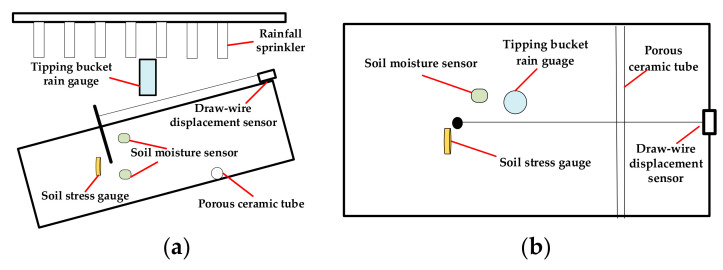
Schematic diagram of sensor installation in the landslide disaster simulation platform. (**a**) Side view of sensor installation schematic; (**b**) Top view of sensor installation schematic.

In the landslide simulation experiment platform, we installed five types of sensors: a tipping bucket rain gauge, a draw-wire displacement sensor, a soil stress gauge, and two moisture content sensors. The installation positions of the sensors are shown in Figure 6.

The locations of the sensors installed in the experiment are as follows:

1. The tipping bucket rain gauge is located in the center of the soil-carrying box, with its opening facing upwards for better rain reception.
2. The position of the draw-wire displacement sensor is in the front third of the soil-carrying box. It monitors the change in soil displacement as the leading edge of the landslide moves.
3. The soil stress gauge is positioned in the front third of the soil-carrying box to monitor the stress changes within the soil at the leading edge of the landslide.
4. The location of the soil moisture sensor for monitoring the shallow moisture content is about 30 cm from the surface, and the location of the soil moisture sensor for monitoring the deep moisture content is about 80 cm from the surface.

Note that the above sensor installation locations are limited by the LSP and are only used as a reference criterion for experiments.

### 3.2. Landslide Data Processing

**Figure 7:**
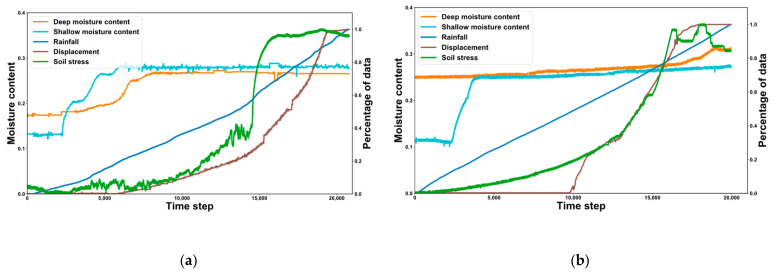
Curve of landslide datasets *L*_1_ and *L*_2_. (**a**) Dataset *L*_1_. (**b**) Dataset *L*_2_.

We carry out two experiments on rainfall-induced landslides and obtain datasets for *L*_1_ and *L*_2_. The rainfall, soil stress, and displacement in the datasets are normalized to obtain the sensor data curves in Figure 7.

The ordinate on the left of Figure 7 is moisture content, and the ordinate on the right is the percentage of data. After a period of time, the moisture content of the soil in the shallow layer begins to rise, and the moisture content of the soil in the deep layer rises in response. The reason why the relationship between the two moisture contents in Figure 7b is not significant is that before rainfall, the deep soil moisture content is high and close to saturation.

The Pearson correlation coefficient method is used to analyze the landslide sensor datasets to analyze the correlation between different types of sensor data.

The Pearson correlation coefficient is suitable for two columns of spaced variables (continuous variables) in a normal distribution. The correlation coefficient and the probability of the correlation can be obtained for two columns of data using Equation (31) when they have the same number of data and correspond to each other.

(31) $$r_{p}=\frac{Cov(X,Y)}{\sigma_{X}\sigma_{Y}}=\frac{\sum_{i=1}^{n}\left(X_{i}-\bar{X}\right)(Y_{i}-\bar{Y})}{\sqrt{\sum_{i=1}^{n}{\left(X_{i}-\bar{X}\right)}^{2}}\sqrt{\sum_{i=1}^{n}{\left(Y_{i}-\bar{Y}\right)}^{2}}}$$

where

$r_{p}$ denotes Pearson correlation coefficient.

*X* represents senor data.

*Y* represents sensor data other than *X*.

$\sigma_{X}$ denotes the standard deviation of *X*.

$\sigma_{Y}$ denotes the standard deviation of *Y*.

The Pearson correlation coefficient ranges between −1 and 1. When the Pearson correlation coefficient is 0, the *X* and *Y* vectors are not correlated. When its value is greater than 0.8, *X* and *Y* are highly correlated.

**Figure 8:**
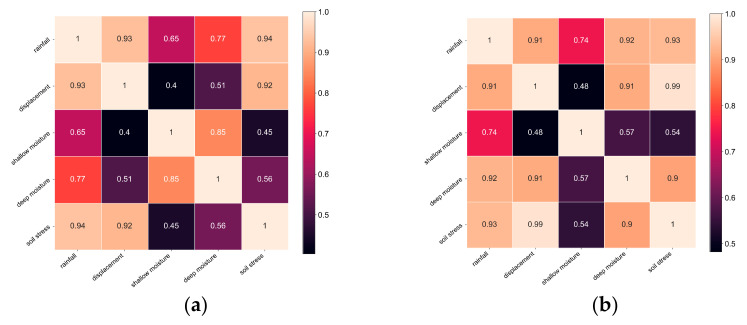
Heatmaps of landslide datasets *L*_1_ and *L*_2_. (**a**) Pearson heatmap of *L*_1_. (**b**) Pearson heatmap of *L*_2_.

We let *X* and *Y* be one of the five types of sensor data, respectively, and the heatmaps are obtained in Figure 8 after the calculation of Equation (31).

In Figure 8a, the rainfall and displacement show a high correlation with the magnitude of soil stress and a moderate correlation with the shallow moisture content and the deep moisture content. The shallow moisture content and the deep moisture content are highly correlated states. The shallow moisture content shows a weak correlation with the displacement amount. Soil stress shows a strong correlation with displacement. In Figure 8b, rainfall displays a strong correlation with displacement, soil stress, and deep moisture content and a moderate correlation with shallow moisture content. The correlation between shallow moisture content and other sensor data is weak. The relationship between the landslide process and different sensor data is analyzed as follows:

1. The amount of rainfall directly affects the moisture content of the shallow soil. Surface water will exist when the surface seepage rate is less than the rainfall.
2. The moisture content of deep soil is significantly higher than that of shallow soil due to groundwater action during the initial stage of rainfall. The moisture content in the deeper layers of the soil would gradually increase as surface water gradually infiltrates into the ground as rainfall continues. However, its moisture content does not exceed the shallow moisture content at this stage. The growth rate of the shallow moisture content would gradually decrease, and the size of the deep moisture content would eventually be approximately equal to the shallow moisture content throughout the entire landslide formation process.
3. The soil stress also varies as the soil layer’s moisture content varies. The shear strength of the soil is characterized by soil stress. The soil stress increases quickly for a while when there is no significant displacement of the surface, after which the surface gradually becomes significantly displaced during the sliding phase. As the soil’s moisture content rises, the clay in the soil softens and loses some of its slip resistance. It also loses shear strength.
4. The soil moisture content tends to become saturated before the landslide body enters the catastrophic slip phase. When the soil stress increases, the landslide body enters the severe sliding stage. When a landslide reaches the severe slip stage, the surface displacement dramatically rises, and erosion-created depressions and gullies start to show up near the body’s front edge.
5. After entering the stabilization stage, the surface displacement of the landslide body no longer increases, but due to the effects of rainfall and groundwater, the surface and underground runoff still play a role in triggering the secondary landslide.

## 4. Experiments and Results

In this section, we describe experiments on landslide warning signals and signal prediction. We present the results of two experiments to demonstrate the effectiveness of En-Attn as well as ATCN in landslide warning signal capture and prediction.

### 4.1. Landslide Hazard Degree and Results

**Figure 9:**
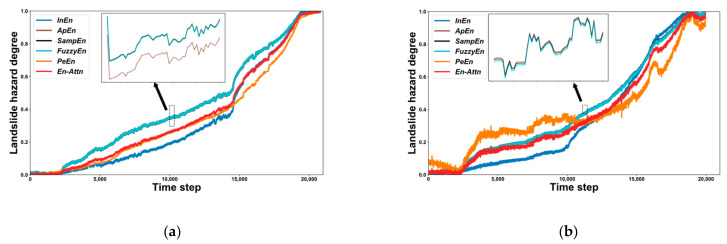
Landslide hazard degree (LHD) of the landslide datasets *L*_1_ and *L*_2_. (**a**) LHD of *L*_1_. (**b**) LHD of *L*_2_.

We apply the En-Attn model to process the landslide datasets *L*_1_ and *L*_2_. Figure 9 illustrates the landslide hazard degree (LHD) obtained by En-Attn as well as the three EWMs. The LHD obtained by all six methods shows an increasing trend, indicating a gradual increase in the characteristics of the hazard level during landslide formation. The LHD ranges from 0 to 1. LHD = 0 means no warning feature, and LHD = 1 means the landslide warning feature is significant and enters a very urgent warning situation. For dataset *L*_1_, the LHD increases gradually, and when the time step is greater than 14,000, the LHD increment rate increases. For dataset *L*_2_, the incremental rate of LHD increases when the time step is greater than 10,000, while the volatility of LHD is greater compared to *L*_1_.

Note that the differences in the LHD obtained by *ApEn*, *SampEn*, and *FuzzyEn* are not significant, and the differences exhibited by the local enlarged image are shown in Figure 8a,b. The reason that only *FuzzyEn* is considered in the En-Attn model and not both *ApEn* and *SampEn* is because the differences between the three methods are not significant.

The single entropy value method is prone to fluctuations in the calculation of LHD, as in the case of *PeEn* in Figure 8b. The LHD obtained by the En-Attn model not only demonstrates landslide warning characteristics but also exhibits better stability and robustness. The En-Attn model overcomes the drawbacks of the single EWM and adapts better to the case of multi-sensor data to evaluate landslide warning features.

### 4.2. Prediction Experiments and Results

We apply the ATCN model to process the landslide datasets *L*_1_ and *L*_2_ and their LHD. The ATCN model is elaborated in Section 2.2. We conducted experiments to test the performance of the ATCN model, comparing long short-term memory neural networks (LSTM) [35], grated recurrent units (GRU) [36], temporal neural networks (TCN) [32,34], convolutional long short-term memory neural networks (ConvLSTM) [37], and dual-stage attention-based recurrent neural networks (DA-RNN) [30]. The metrics [2] for evaluating the performance are root mean square error (RMSE), mean absolute error (MAE) and mean absolute percent error (MAPE), and the specific equations are shown in Equations (32)–(34).

(32) $$\mathrm{MAE}=\frac{1}{N}\sum_{t=1}^{N}\left|{\hat{y}}_{t}-y_{t}\right|$$

(33) $$\mathrm{RMSE}=\sqrt{\frac{1}{N}\sum_{t=1}^{N}{\left({\hat{y}}_{t}-y_{t}\right)}^{2}}$$

(34) $$\mathrm{MAPE}=\frac{100\%}{N}\sum_{t=1}^{N}\left|\frac{{\hat{y}}_{t}-y_{t}}{y_{t}}\right|$$

where

*N* is the total number of test data.

$y_{t}$ is the true value at the *t*th time step.

${\hat{y}}_{t}$ is the predicted value at the *t*th time step.

The model tests are divided into two types of sliding windows, “100-10” and “100-50”, which reflect different input data lengths and prediction lengths. The hyperparameters of the TCN and ATCN models are set as follows: filters = 32, batch size = 128, kernel size = 8, where the activation function of the attention mechanism is *Softmax*. The hyperparameters of the LSTM and GRU models are set as follows: the number of units is 16. The activation function is *ReLU*, the optimization algorithm is *Adam*, the initial learning rate is 0.001, and the learning rate can be adjusted according to the loss function subsequently. The hyperparameter experiments of ATCN are shown in Appendix A. All models are run 20 times, and the predicted values are obtained after testing the datasets *L*_1_ and *L*_2_. The average values of RMSE, MAE, and MAPE are shown in Table 1 and Table 2.

Table 1 and Table 2 demonstrate the RMSE, MAE, and MAPE of ATCN and its counterparts. Table 1 shows that the RMSE, MAE, and MAPE metrics of ATCN are lower for dataset *L*_1_, which implies better performance of ATCN.

The ATCN outperforms other models in the prediction of LHD. Compared with the TCN model, the RMSE, MAE, and MAPE of ATCN decreased by 55.60%, 52.13%, and 51.17%, respectively, with the sliding window set to “100-10”. The ATCN can effectively capture the characteristics of landslide prediction. The ATCN also outperforms other models when the sliding window is “100-50”. In comparison to the TCN model, the performance of the three metrics is decreased by 43.30%, 35.63%, and 34.24%, respectively. The poor performance is due to the absence of attention mechanisms in the LSTM, GRU, and ConvLSTM, as well as the insignificant features obtained from the complex landslide sensor signals.

Figure 2 displays the metrics for dataset *L*_2_, which is similar to dataset *L*_1_. The classical recurrent neural network models, LSTM and GRU, performed poorly because the predictive properties shown by the sensor data in dataset *L*_2_ are not obvious. The performance of DA-RNN and ATCN with the addition of the attention mechanism is outstanding. The three metrics of ATCN are decreased by 33.74%, 30.15%, and 29.06%, respectively, in comparison to DA-RNN when the sliding window is set to “100-10”. The three metrics of ATCN are decreased by 35.97%, 35.44%, and 35.10%, respectively, compared to DA-RNN when the sliding window is set to “100-50”.

Comparing the model performance with different prediction lengths, it can be seen that the shorter the prediction length, the smaller the performance metrics, and the better the prediction effect. When the prediction length is long, the attention mechanism captures the long-term dependency characteristics more and more prominently, and the performance of DA-RNN and ATCN with the attention mechanism is better than the other models. Comparing the DA-RNN and ATCN models, ATCN has better prediction results and stable performance when the sliding windows are “100-10” and “100-50”. The ATCN model has the lowest error and the best prediction, as seen in Table 1 and Table 2. The two sliding windows can be compared to demonstrate that the model’s error increases with prediction length. ATCN’s prediction accuracy is greater.

## 5. Discussion and Conclusions

This work adopts the attention mechanism to integrate the multi-entropy values to capture the landslide warning signals and explores the ATCN to realize landslide hazard prediction. Compared with its counterparts, our model has the characteristics of higher accuracy. Compared with current landslide hazard prediction methods, our methods have the following characteristics:

1. Exploring deep learning algorithms combined with big landslide data is an extension of deep learning application scenarios. This model uses a simple attention mechanism combined with a temporal convolutional neural network. Although this model is simple, its prediction effect is better than other complex deep learning models.
2. Effective landslide hazard capture. In the traditional sense, the capture of rainfall-induced landslide hazards is either directly replaced by the landslide displacement or only a single EWM is used to realize the signals capture. The model uses the attention mechanism to integrate a variety of EWMs, and the obtained landslide warning signals are more reliable.
3. Note that our model cannot be adapted for landslide hazard prediction with a small amount of data, as massive data is the basis of our model.

In the future, we intend to design a software system that integrates the algorithms for actual landslide sites. Further, we intend to consider different types of sensor data because more kinds of sensor data represent more comprehensive landslide disaster information. Furthermore, we plan to consider the sensor data of the landslide simulation platform in relation to soil thickness. We use landslide simulation experiments in this study. However, we could not achieve the exact same processes in the laboratory as in nature. For example, simulating different soil layers, which would take millions of years to form in nature. Our future research work will take into account multiple natural environmental factors to improve the experimental setup, including slope angle and dynamics of water extinction.

## Figures and Tables

**Table 1:** Comparison of LHD prediction effects of different models for dataset *L*_1_.

Model	Metric	Size of Sliding Window	
		100-10	100-50
LSTM	RMSE	0.04973	0.05987
	MAE	0.03483	0.03988
	MAPE (%)	3.45876	4.48301
GRU	RMSE	0.04296	0.11422
	MAE	0.02916	0.10989
	MAPE (%)	3.21155	4.70642
ConvLSTM	RMSE	0.01511	0.02480
	MAE	0.01162	0.02307
	MAPE (%)	1.31189	2.70816
DA-RNN	RMSE	0.02606	0.02044
	MAE	0.01825	0.01590
	MAPE (%)	1.96037	1.68211
TCN	RMSE	0.02009	0.03222
	MAE	0.01500	0.02192
	MAPE (%)	1.68965	2.42844
ATCN	RMSE	0.00892	0.01827
	MAE	0.00718	0.01411
	MAPE (%)	0.82503	1.59699

**Table 2:** Comparison of LHD prediction effects of different models for dataset *L*_2_.

Model	Metric	Size of Sliding Window	
		100-10	100-50
LSTM	RMSE	0.04465	0.10245
	MAE	0.03571	0.09849
	MAPE (%)	3.74129	6.12409
GRU	RMSE	0.03632	0.06781
	MAE	0.02316	0.05799
	MAPE (%)	2.41790	4.88399
ConvLSTM	RMSE	0.02937	0.05297
	MAE	0.02369	0.03579
	MAPE (%)	2.56583	3.82107
DA-RNN	RMSE	0.01633	0.02966
	MAE	0.01360	0.02266
	MAPE (%)	1.44912	2.38209
TCN	RMSE	0.02540	0.03209
	MAE	0.02059	0.02687
	MAPE (%)	2.16727	2.84709
ATCN	RMSE	0.01082	0.01899
	MAE	0.00950	0.01463
	MAPE (%)	1.02798	1.54598

## Data Availability

Not applicable.

## Appendix A. Hyperparameter Experiments of the ATCN

The hyperparameters in ATCN can directly affect the high performance of the landslide prediction model. The kernel size, filters, and training batch size in the model has a large impact on ATCN. With dataset *L*_2_, performance comparison experiments are carried out on the kernel sizes, filters, and batch sizes in the ATCN model. The comparison metrics are RMSE, MAE, and MAPE, and the experiments of each hyperparameter are repeated 20 times, and the mean values of the 20 experiments are counted. The statistical results are shown in Table A1, Table A2 and Table A3.

**Table A1:** Comparison of different batch sizes in the ATCN model.

Batch Size	Metric	Size of Sliding Window	
		100-10	100-50
16	RMSE	0.01452	0.01928
	MAE	0.01325	0.01723
	MAPE (%)	1.63992	1.86792
32	RMSE	0.01213	0.01989
	MAE	0.01069	0.01907
	MAPE (%)	1.08400	2.37950
64	RMSE	0.01614	0.01734
	MAE	0.01609	0.01609
	MAPE (%)	1.11208	1.73150
128	RMSE	0.00954	0.01929
	MAE	0.00943	0.01606
	MAPE (%)	1.00213	0.91316
256	RMSE	0.01619	0.01892
	MAE	0.01825	0.01838
	MAPE (%)	2.19243	1.99731

**Table A2:** Comparison of different filters in the ATCN model.

Filter	Metric	Size of Sliding Window	
		100-10	100-50
4	RMSE	0.01674	0.01937
	MAE	0.01531	0.01334
	MAPE (%)	1.64269	1.56591
8	RMSE	0.01016	0.01102
	MAE	0.01158	0.00934
	MAPE (%)	1.31589	1.13547
16	RMSE	0.01023	0.01803
	MAE	0.01709	0.00949
	MAPE (%)	1.82595	1.86010
32	RMSE	0.01953	0.01597
	MAE	0.01897	0.01504
	MAPE (%)	1.07723	1.88453
64	RMSE	0.11779	0.01696
	MAE	0.01085	0.01360
	MAPE (%)	1.42817	1.63355

**Table A3:** Comparison of different kernel sizes in the ATCN model.

Kernel Size	Metric	Size of Sliding Window	
		100-10	100-50
4	RMSE	0.01148	0.01582
	MAE	0.01810	0.01442
	MAPE (%)	1.47336	1.54591
8	RMSE	0.00984	0.01074
	MAE	0.09313	0.00943
	MAPE (%)	1.39457	1.03825
16	RMSE	0.00949	0.00965
	MAE	0.00809	0.00807
	MAPE (%)	0.89151	0.98417
32	RMSE	0.10553	0.00963
	MAE	0.01805	0.00909
	MAPE (%)	1.37068	1.08417
64	RMSE	0.00959	0.10772
	MAE	0.01168	0.10620
	MAPE(%)	1.21431	1.05872

Table A1 shows the metrics of ATCN for different batch sizes tested with kernel size = 16, filters = 8. The results in Table A1 show that the RMSE, MAE, and MAPE metrics of the model for both sliding window cases are the smallest for batch size = 128. Table A2 provides the metrics of ATCN with different filters tested for batch size = 128 and kernel size = 16. The sliding window “100-50” model exhibits the smallest RMSE, MAE, and MAPE metrics when filter = 8, according to Table A2. Table A3 demonstrates the metrics of ATCN for different kernel sizes with batch size = 128 and filters = 8. The results in Table A3 demonstrate that for the sliding window “100-10” with kernel size = 16, the RMSE, MAE, and MAPE metrics are minimum. The smallest MAE and MAPE metrics are for the sliding window “100-50” with kernel size = 16. The optimal combination of hyperparameters for the ATCN model is batch size = 128, kernel size = 16, and filters = 8.

Note that our model code runs on Windows 10, NVIDIA GeForce GTX 1650 GPU, and the deep learning framework is TensorFlow 2.6.0.
